# Supplementary material for: Complex‐centric proteome profiling by SEC‐SWATH‐MS
Source: Mol Syst Biol. 2019 Jan 14;15(1):e8438. doi: 10.15252/msb.20188438 (PMC6346213; doi:10.15252/msb.20188438)
Supplement: Supplementary file 8 — Dataset EV7 [file MSB-15-e8438-s008.zip › feature_plots_string/Q8N1F7.pdf]

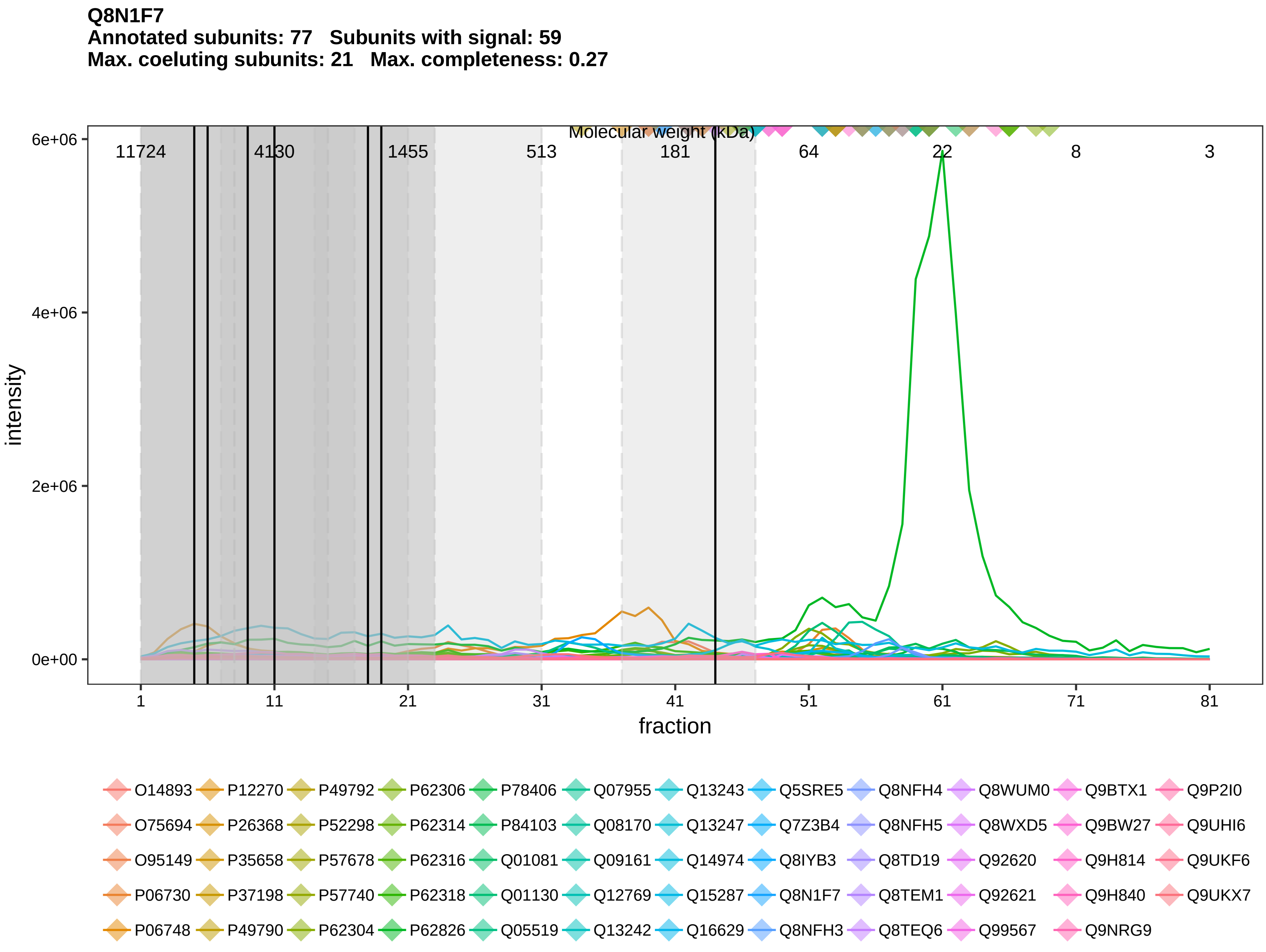

Q8N1F7
Annotated subunits: 77 Subunits with signal: 59
Max. coeluting subunits: 21 Max. completeness: 0.27
Molecular weight (kDa)
11724
4130
1455
513
181
64
22
8
3
intensity
6e+06
4e+06
2e+06
0e+00
fraction
1
11
21
31
41
51
61
71
81
O14893
O75694
O95149
P06730
P06748
P12270
P26368
P35658
P37198
P49790
P49792
P52298
P57678
P57740
P62304
P62306
P62314
P62316
P62318
P62826
P78406
P84103
Q01081
Q01130
Q05519
Q07955
Q08170
Q09161
Q12769
Q13242
Q13243
Q13247
Q14974
Q15287
Q16629
Q5SRE5
Q7Z3B4
Q8IYB3
Q8N1F7
Q8NFH3
Q8NFH4
Q8NFH5
Q8TD19
Q8TEM1
Q8TEQ6
Q8WUM0
Q8WXD5
Q92620
Q92621
Q99567
Q9BTX1
Q9BW27
Q9H814
Q9H840
Q9NRG9
Q9P2I0
Q9UHI6
Q9UKF6
Q9UKX7
